# Histopathological Characterization of a Series of Oral Basaloid Squamous Cell Carcinoma

**DOI:** 10.1155/2023/6036567

**Published:** 2023-04-10

**Authors:** Neetu Jain, Toniya Raut, Shashi Keshwar, Ashish Shrestha, Mehul Rajesh Jaisani, Deepak Paudel

**Affiliations:** ^1^Department of Oral Pathology, College of Dental Surgery, B.P. Koirala Institute of Health Sciences, Dharan, Nepal; ^2^Department of Oral and Maxillofacial Surgery, College of Dental Surgery, B.P. Koirala Institute of Health Sciences, Dharan, Nepal; ^3^Department of Otolaryngology & HNS, B.P. Koirala Institute of Health Sciences, Dharan, Nepal

## Abstract

Basaloid squamous cell carcinoma (BSCC) is a rare, distinctive, and aggressive variant of squamous cell carcinoma (SCC) primarily seen in the upper aerodigestive tract with epiglottis, soft palate, and base of the tongue being site of high preference in head and neck region. It differs from conventional SCC histologically and immunologically, is most frequently found in males in their sixth and seventh decades, and is frequently linked to alcohol and tobacco use. High stage disease with distant metastases, a high recurrence rate, and a dismal prognosis is how BSCC typically manifests. In the present article, we report four cases of BSCC.

## 1. Introduction

Basaloid squamous cell carcinoma, or BSCC, is a rare form of squamous cell carcinoma (SCC) with elements of both basaloid and conventional SCC [1, 2]. It was first described in the head and neck region by Wain et al. [1]. As per WHO classification of head and neck tumors 2017, it is categorized as malignant surface epithelial tumors and defined as clinically unfavorable variant of SCC composed of a prominent basaloid component and with evidence of squamous cell differentiation [3]. Similar to conventional SCC, BSCC has a strong connection to alcohol and tobacco [4]. It typically affects men over the age of sixth decade [5].

The malignance has been suggested to have initiated from a totipotent primitive cell in the basal cell layer of the surface epithelium or in the proximal duct of secretory glands, though the histogenesis of BSCC is still poorly understood [6]. Histologically, BSCC is characterized by a dimorphic pattern, with basal cells arranged in sheets and lobules giving a jigsaw puzzle appearance exhibiting nuclear pleomorphism, hyperchromatic, and indistinct cytoplasm associated with the squamous portion with cystic spaces containing periodic acid Schiff (PAS) material and having myxoid material [7]. Other common histological features include areas of comedo necrosis and overlying epithelium with carcinomatous changes [8].

We present four BSCC cases from dorsum of tongue, floor of the mouth, uvula, and lower labial vestibule.

## 2. Case Presentations

### 2.1. Case Report-I

A 32-year-old female patient reported with a history of wound on dorsum of tongue since 6 months. The lesion was painful, with burning sensation on consumption of food, erythematous, round, heterogeneous, irregular with insidious onset, and progressive in nature. Patient also gave a history of betel nut chewing since 15 years along with a past history of SCC on right buccal mucosa for which she underwent surgery and radiotherapy 6 years back.

**Figure 1:**
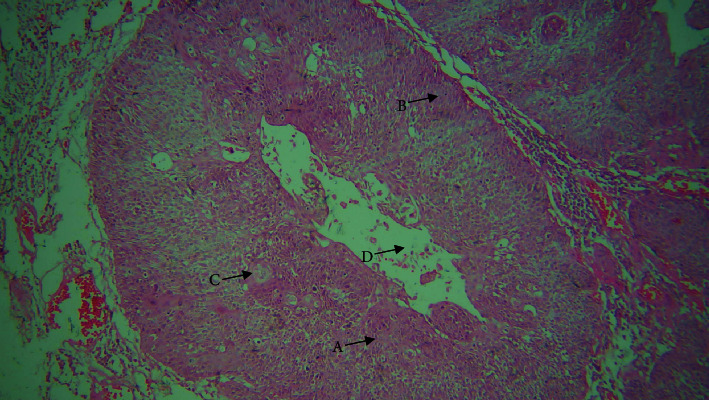
10× view. A: squamous cells; B: basaloid cells; C: keratin pearl; and D: area of comedo necrosis.

**Figure 2:**
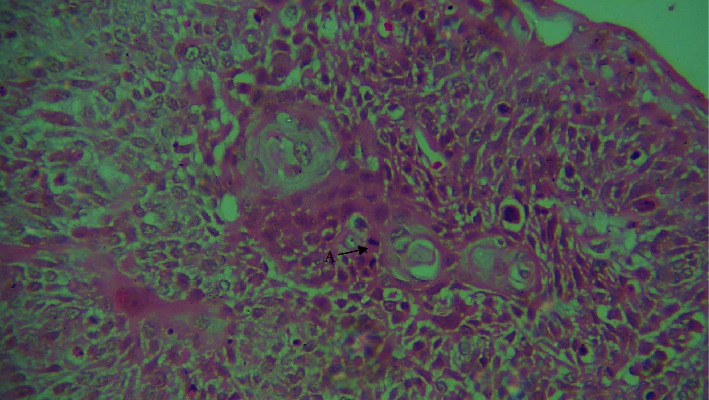
40× view. A: mitotic figure.

On histological inspection, the lesion revealed dysplastic epithelium that had invaded the severely inflamed connective tissue in the form of duct like structures. Within these duct like structures, basaloid appearing cells with hyperchromatic nuclei and sparse cytoplasm having palisading arrangements intermixed with squamous cells, keratin pearl production, mitotic figures, and some clear cells were evident. Comedo necrosis was observed in the center of these lobules (Figures 1 and 2). A histopathological diagnosis of BSCC was made concurrently with the findings mentioned above.

### 2.2. Case Report-II

**Figure 3:**
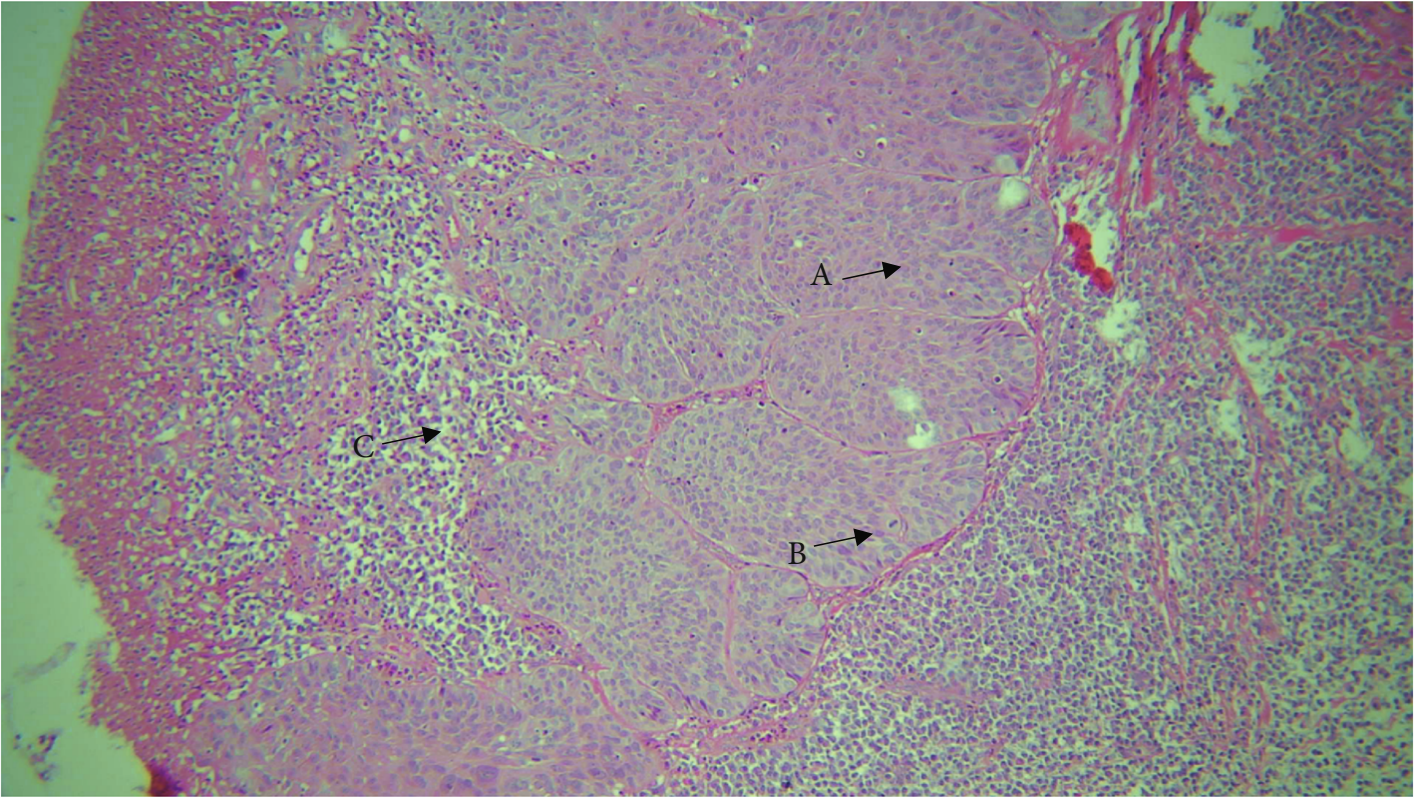
10× view. A: basaloid cells; B: mitotic figure; and C: inflammatory cells.

**Figure 4:**
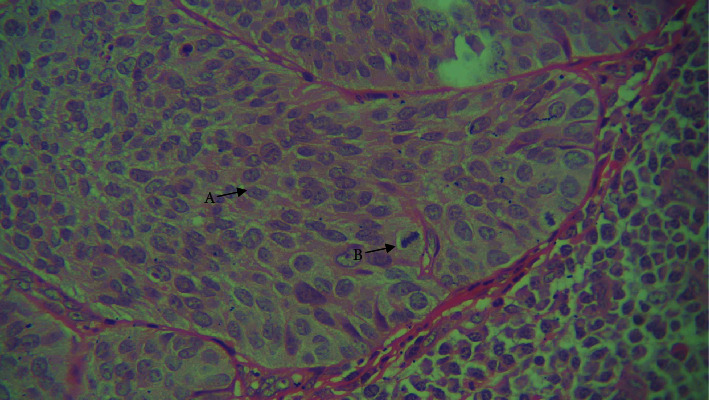
40× view. A: basaloid cells; and B: mitotic figure.

A 64-year-old female patient reported with an ulceroproliferative growth in the floor of the mouth without any significant habit history. Incisional biopsy of the section revealed dysplastic stratified squamous epithelium along with ulcerated areas. The underlying connective tissue revealed infiltrated basaloid cells arranged in the form of lobules with hyperchromatic nuclei and scanty cytoplasm in association with polygonal squamous cells having abundant cytoplasm. Numerous abnormal mitotic figures were evident. The stroma surrounding the tumor was dense with chronic inflammatory cell infiltrate (Figures 3 and 4). A histopathological diagnosis of BSCC was made in accordance with the above mentioned features.

### 2.3. Case Report-III

**Figure 5:**
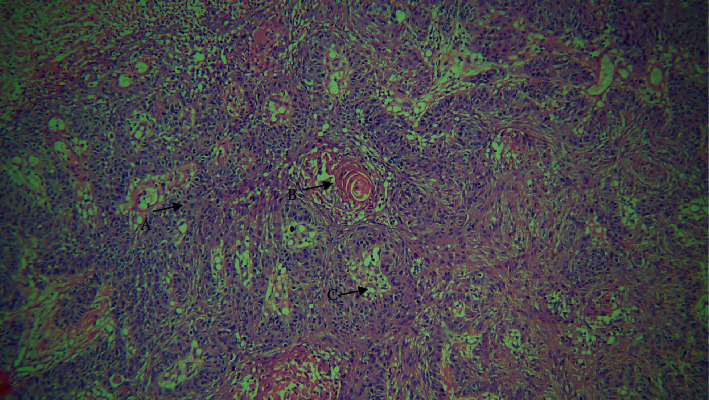
4× view. A: basaloid cells; B: keratin pearl; and C: comedo necrosis.

**Figure 6:**
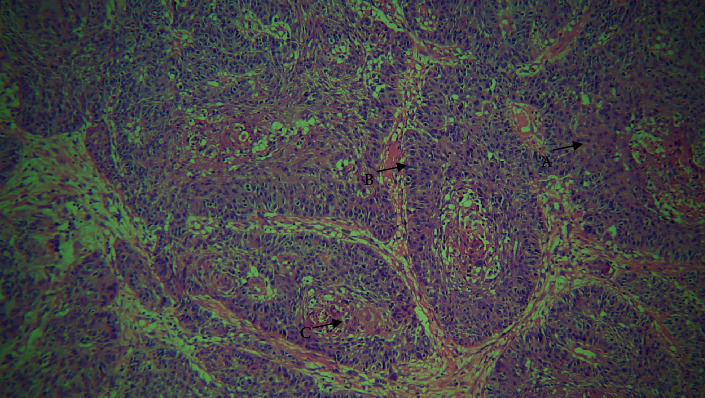
10× view. A: squamous cells; B: basaloid cells with peripheral palisading; and C: comedo necrosis.

A paraffin embedded tissue block of a 51-year-old female referral patient with a history of smoking since 15 years was received. The patient had polypoid strawberry uvula. Upon histological examination, the section revealed overlying dysplastic epithelium exhibiting cellular and nuclear pleomorphism, nuclear hyperchromatism. Within the connective tissue, the tumor islands and lobules appeared biphasic with central small cuboidal cells with dark, hyperchromatic nuclei, and scanty cytoplasm resembling basaloid cells, whereas the peripheral columnar cells exhibited a palisading pattern. Comedo necrosis in the center of these structures was also evident. Clear cells were also apparent in some areas (Figures 5 and 6). Concurrent with the aforementioned observations, histopathological diagnosis of BSCC was made.

### 2.4. Case Report-IV

**Figure 7:**
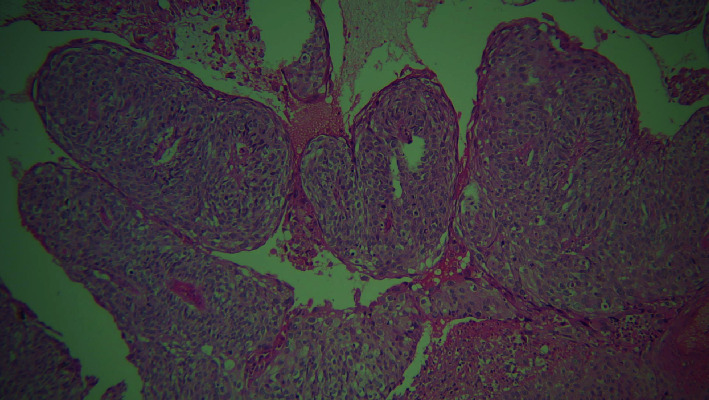
4× view: islands of basaloid cells intermixed with squamous cells.

**Figure 8:**
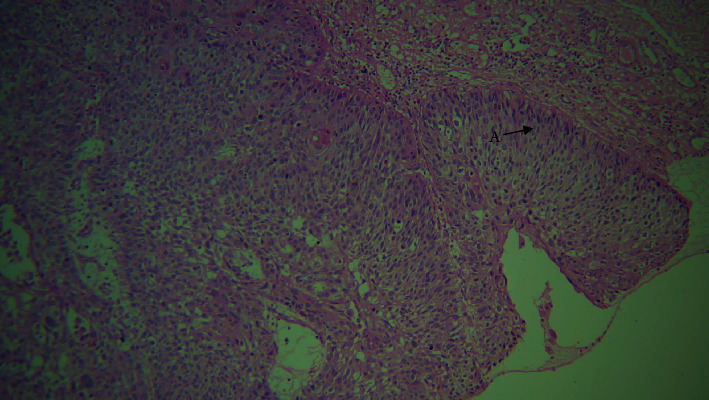
10× view. A: basaloid cells with peripheral palisading.

A 68-year-old male patient reported with a history of swelling on upper half of the chin. On extra-oral examination, there was a single, well defined swelling of size 6 × 4 cm^2^, roughly oval in shape, and tender on palpation with normal overlying skin. Localized rise of temperature was present over the swelling but no discharge was seen. Upon intra-oral examination, indurated ulcerated lesion was evident in the lower labial vestibule. Patient also gave a history of tobacco chewing placed in the region of lower labial vestibule at least 20 times a day since 50 years along with smoking one packet of cigarette per day. Histopathological examination of the incisional biopsy was done, which revealed dysplastic epithelium invading the connective tissue stroma in the form of islands, lobules, and duct like structures having a biphasic appearance with one group of cells, round to oval in shape with scanty cytoplasm, and hyperchromatic nuclei resembling basaloid cells, whereas another population of cells resembled squamous cells. Additionally, peripheral palisading of cells, comedo necrosis, numerous mitotic figures, and keratin pearl formation was also evident (Figures 7 and 8). Based on the above mentioned findings, the case was diagnosed as BSCC.

## 3. Discussion

BSCC is a rare subtype of SCC. Approximately, 1% of SCC are BSCC. BSCC is a highly malignant, biomorphic, and extremely aggressive tumor [9]. In comparison to stage-matched conventional SCC, which frequently includes local recurrences and regional and distant metastases, it is generally acknowledged that BSCC in the head and neck region tends to have an aggressive clinical history [1].

BSCC is mostly seen in older males who consume large amounts of alcohol and smoke. In our case reports, three cases were reported in elderly patients of age 51, 64, and 68 years, whereas in one of the case, it was seen to arise at quite an early age of 32 years. Among the reported cases, one presented with a history of smoking, another with a history of betel nut chewing, one with a history of both tobacco chewing and smoking, thus establishing the relationship of the lesion with these habits, whereas one of the patient did not reveal any habit history. As per literature, BSCC is more common in males, but in three of our reported cases, BSCC was seen in females [5].

In order to explain the histogenesis of BSCC, various theories have been postulated. The proximal ducts of the minor salivary glands, the totipotent primitive cells of the surface epithelium, or the tissues of the primitive aerodigestive tract have all been suggested as their sources. Some authors also consider that “basaloid pattern arising anywhere in the body signifies an attempt at glandular differentiation” [10]. It is interesting that Wain et al. [1] have suggested that this might be a hybrid tumor with two independent morphological components or perhaps a carcinoma with adenoid cystic differentiation [1].

The tumor appears macroscopically as an ulcerated exophytic firm mass. The term basaloid describes conglomerated cells that typically exhibit an elevated nuclear/cytoplasmic ratio, sparse amphiphilic cytoplasm, and oval, hyperchromatic nuclei without noticeable nucleoli histologically. Nuclear pleomorphism and mitotic figures are frequently seen. Cords, nests, islands, and lobules are castoff to organize the basaloid components [11]. Areas of comedo necrosis can be evident as area of coagulative necrosis within central areas of tumor lobules might be due to deficient nutritional and blood supply to the central cells of the rapidly dividing basaloid cells. Also, localized keratinization is seen in tumor islands [12]. In all the above reported cases, we observed the basaloid cells with an increased nuclear/cytoplasmic ratio, scant amphiphilic cytoplasm, and oval, hyperchromatic nuclei without prominent nucleoli. Mitotic figures and nuclear pleomorphism were observed in numerous areas. The basaloid components were arranged in cords, nests, islands, and lobules. Tumor islands exhibit basaloid cells with areas of comedo necrosis in two cases, whereas in one of the case, comedo necrosis was not evident. Focal keratinization was evident in all the three cases. The presence of spindle cells with elongated nuclei and scant eosinophilic cytoplasm at the periphery of the nest of basaloid cells giving a palisading appearance was also evident.

Despite the reported distinctive histopathologic pattern, BSCC can be mistaken for adenoid cystic carcinoma (solid type) (ACC), basal cell adenosquamous carcinoma, small cell undifferentiated carcinoma, salivary duct carcinoma, neuroendocrine carcinoma, and basal cell ameloblastoma [10].

Oral squamous cell carcinoma (OSCC) can be distinguished from BSCC despite the clinical similarities between OSCC and BSCC, because it lacks the stromal hyalinization and cystic areas filled with Periodic acid–Schiff (PAS) stain-positive material that BSCC exhibits [12].

Similar to BSCC the basaloid-appearing solid type of ACC is composed of sheets or islands of tumor cells with an angulated hyperchromatic nucleus and sparse cytoplasm. However, it can be distinguished from BSCC by the absence of squamous differentiation, the presence of myoepithelial cells, and perineural invasion. Positive immunostaining of the ACC with smooth muscle actin (SMA) and the absence of SMA in the BSCC aid in distinguishing the cell type [13].

Adenosquamous carcinoma can be distinguished from BSCC by having mucin positive and genuine ductal acinar differentiation. Basal cell adenocarcinoma lacks focal necrosis and squamous differentiation, which are typically present in BSCC. Salivary duct carcinomas can be distinguished from BSCC by their eosinophilic cytoplasm and irregularly formed cystic areas bordered with papillary projections [14].

It is crucial to keep in mind that neuroendocrine carcinoma differs from BSCC, in that, it rarely connects to the surface mucosa and exhibits the typical nuclear molding, crushing artifact, lack of stromal mucinous myxoid alterations, and hyalinosis. Additionally, chromogranin, synaptophysin, and glial fibrillary acid protein are also negative in BSCC [14].

When odontogenic epithelium is present and there is no central comedo necrosis or squamous component, basal cell ameloblastoma can be distinguished from BSCC [15]. Histopathology of basal cell ameloblastomas resembles BCC, but utilizing Ber-EP4, an immunohistochemical distinction can be made [16]. Despite of sharing a histological similarity with BSCC in the oral cavity, basal cell carcinoma is incredibly rare [14].

Numerous recent studies have statistically shown that BSCC seems to follow a completely different clinical course depending on the tumor site. Oropharyngeal BSCC generally shows a better prognosis, whereas laryngeal and non-oropharyngeal BSCC shows a poorer survival. Some authors have also recently proposed a favorable prognosis in Human papilloma virus (HPV) induced BSCC. So it is important not to confuse cases affecting the oral cavity from those affecting the oropharyngeal region [17, 18].

The present case series revealed the typical histologic picture of BSCC. The parts of dysplastic squamous epithelium infiltrating into connective tissue were evident, and the tumor cells presented a basaloid appearance with peripheral palisading, comedo necrosis, and occasional squamous differentiation, which prompted us to give a final diagnosis of BSCC. Although the clinical diagnosis is simply that of an oral SCC, it should be a routine to diagnose the case histologically as any of its variants. The diagnosis of these cases as BSCC is essential as the lesion has a different clinical course, prognosis, and treatment aspects when compared to the conventional oral SCC.

## 4. Conclusion

The clinical aggressiveness of BSCC compared to the conventional SCC is still controversial. However, it has been correlated that cases with pure basaloid form and comedo necrosis have more probability for distant metastasis might be owing to the high mitotic capacity of undifferentiated basaloid cells. Patients usually present at the advanced stage, which could be a reflective of aggressiveness and poor prognosis. So it is crucial to make a clear and prompt diagnosis in order to effectively treat BSCC.

## Data Availability

Data supporting this research article are available from the corresponding author or first author on reasonable request.
